# Sperm cryopreservation and in vitro fertilization techniques for the African turquoise killifish *Nothobranchius furzeri*

**DOI:** 10.1038/s41598-021-96383-8

**Published:** 2021-08-25

**Authors:** Luca Dolfi, Tsz Kin Suen, Roberto Ripa, Adam Antebi

**Affiliations:** 1 Max Planck Institute for Biology of Ageing, Cologne, Germany; 2 Cologne Excellence Cluster On Cellular Stress Responses in Aging Associated Diseases (CECAD), University of Cologne, Cologne, Germany

**Keywords:** Biological techniques, Ageing, Embryology, Reproductive biology

## Abstract

Over the last decade, the African turquoise killifish, *Nothobranchius furzeri,* has emerged as an important model system for the study of vertebrate biology and ageing. Propagation of laboratory inbred strains of *Nothobranchius furzeri*, such as GRZ, however, can pose challenges due to the short window of fertility, the efforts and space requirements involved in continuous strain maintenance, and the risks of further inbreeding. The current method for long term strain preservation relies on arrest of embryos in diapause. To create an alternative for long term maintenance, we developed a robust protocol to cryopreserve and revive sperm for in vitro fertilization (IVF). We tested a variety of extender and activator buffers for sperm IVF, as well as cryoprotectants to achieve practical long-term storage and fertilization conditions tailored to this species. Our protocol enabled sperm to be preserved in a cryogenic condition for months and to be revived with an average of 40% viability upon thawing. Thawed sperm were able to fertilize nearly the same number of eggs as natural fertilization, with an average of ~ 25% and peaks of ~ 55% fertilization. This technical advance will greatly facilitate the use of *N. furzeri* as a model organism.

## Introduction

Over the last few years, the African killifish *Nothobranchius furzeri* has emerged as an important model system for the study of vertebrate biology. *Nothobranchius furzeri*’s life cycle is characterized by a fast growth rate, reaching sexual maturity by 4–5 weeks, and a maximum lifespan of 6.5–7 months in the laboratory strain GRZ-AD^1,2^, making this fish among the shortest-lived vertebrate species bred in captivity and providing a unique platform for the rapid exploration of ageing and age-associated diseases^3^.

Rapid growth, maturation and ageing carry drawbacks, however, which make maintenance in captivity of *N. furzeri* laboratory strains challenging. The short life cycle leads to a swift passage of generations, which makes it difficult to maintain a primary founder genotype and prevent excessive inbreeding^4,5^. In laboratory inbred strains, fertility is typically limited to the 5th–20th week and maximal between the 6th–11th weeks^6,7^ of life, giving a narrow breeding window, though in longer-lived outbred stocks the breeding window can be significantly greater^8^. Importantly, the success of transposon-mediated transgenesis^9^ and CRISPR-mediated mutagenesis^10^ in *Nothobranchius* means that more genetically engineered lines require constant maintenance and space usage. Breeding to preserve a line takes significant effort and carries the risk of accident or infection that can result in strain loss. Furthermore, *N. furzeri* husbandry requires considerable space, since they are often optimally grown when singly housed because of fish-to-fish aggression and food competition^7^.

Given these constraints, it is essential to develop protocols to maintain stocks without constant breeding. Currently, the main way to maintain strains is through embryonic diapause, a state of arrested development^2^. To date, it can be challenging to consistently induce and release diapause in a controlled, synchronized manner from inbred laboratory stocks^11,12^, though recently vitamin D has been shown to regulate diapause in other killifish species^11^. Moreover, embryos in diapause need sporadic but periodic maintenance, since the medium and substrate must be checked, cleaned and sometimes changed^7^. As the number of strains and lines increase, this method becomes untenable at larger scales.

To solve these problems, fish researchers rely on sperm cryopreservation and in vitro fertilization techniques. Through cryopreservation, fish genetic pools can be stored in sperm banks with minimum maintenance effort for years. Upon revival, thawed sperm can usually fertilize eggs with a success rate from 10 to 80%^13^. Specific protocols have been established to preserve and activate the sperm of various species used in aquaculture or research^14^. To date, however, there is no established protocol available for sperm cryopreservation and in vitro fertilization for killifish species. Here we developed a feasible protocol for killifish sperm cryopreservation and in vitro fertilization. Our method should facilitate the husbandry and the usefulness of *N. furzeri* as a model organism for research.

## Results

### Extender and activator

IVF protocols from other fish species make use of an “extender” and an “activator” solution. The extender is usually a saline-buffered solution mixed with the extracted sperm that keeps them stable and inactive, and is typically isotonic or slightly hypertonic to the gonad^13,15^. By contrast, the activator is a low molality solution, which when added to the sperm-extender solution, initiates sperm activity and movement. In some cases, the activator is simply a dilution of the extender. Once activated, the sperm exhibit a short period of motility (30 s to 5 min), depending on the species^16^. Zebrafish and medaka protocols for sperm preservation and activation typically use Hank's Balanced Salt Solution (HBSS), Buffered sperm motility-inhibiting solution (BSMIS) or Fetal bovine serum (FBS) as extender. A dilution of these solutions or the addition of another composite salt, such as Instant Ocean or Iwamatsu solution, trigger activation^14,17,18^.

**Figure 1:**
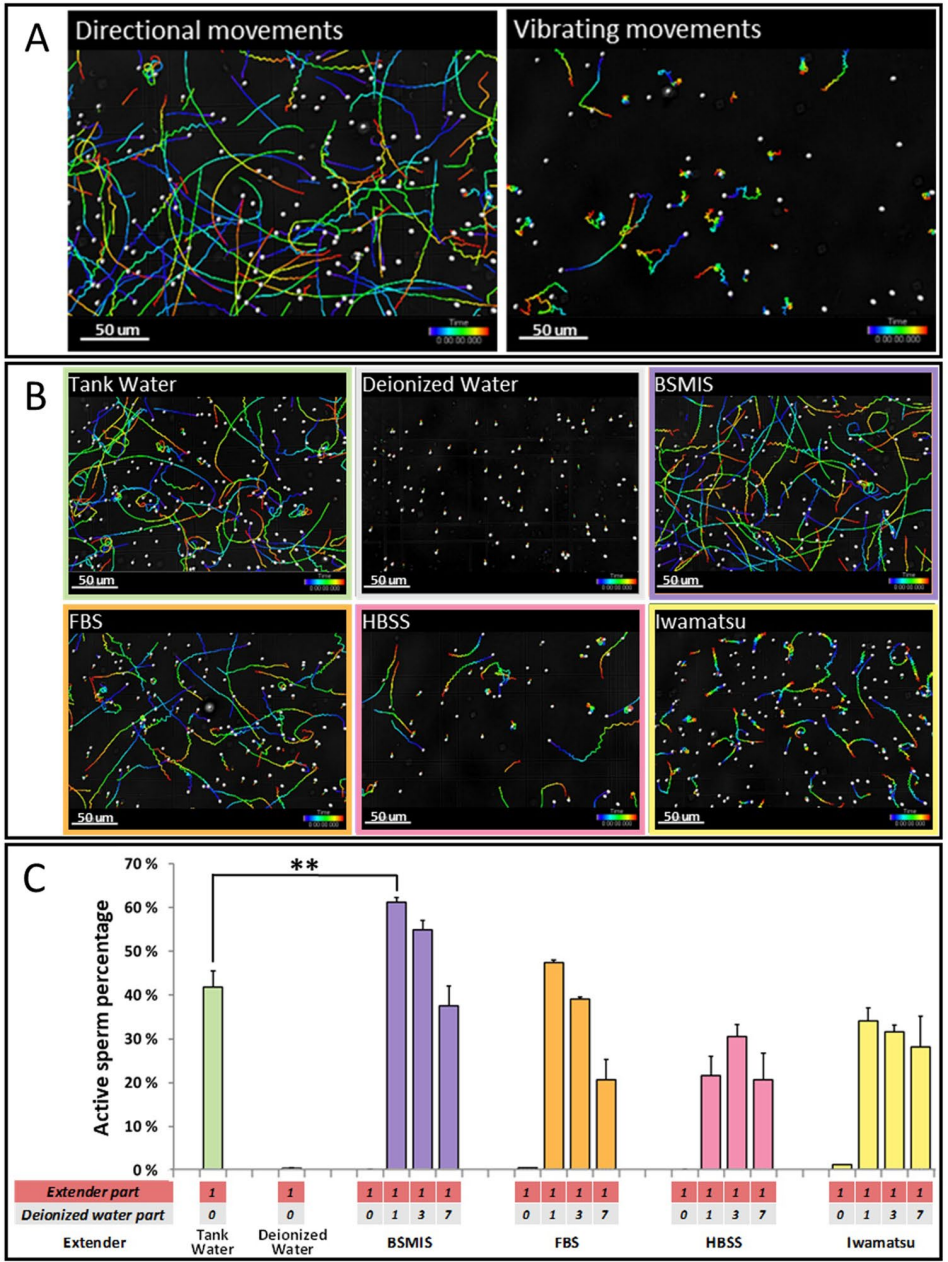
Fresh sperm activation in different buffers. (**A**) Motility tracing of different types of sperm movement that occur after activation, resulting in proper directional movements (left) or erratic inefficient vibrating movements (right). (**B**) Representative images of sperm movement in samples activated by 1:1 dilution of the indicated buffers. (Images in A and B were exported from Imaris, version 8.1, https://imaris.oxinst.com/). (**C**) Quantification of sperm activation in different buffers, calculated as the average number of active sperm divided by total sperm among biological replicates. Asterisks indicate solutions that are significantly more effective than tank water in activating sperm based on a two-way ANOVA post hoc Dunnett’s test. *p < 0.05, **p < 0.01, ***p < 0.001. N = 3 biological replicates for each condition.

Our first goal, therefore, was to test sperm activation using these known solutions. We therefore immersed the gonads in various solutions, testing tank water, deionized water, BSMIS, FBS, HBSS and Iwamatsu solution at different dilutions and thus molalities (Fig. 1).

Tank water, the natural medium where fish spawn, was able to activate ~ 40% of the sperm. Deionized water was able to trigger an initial activation, which faded within seconds (data not shown), resulting immediately afterwards in vibrating or immobile sperm. Stock solutions of BSMIS, FBS, HBSS and Iwamatsu solutions kept the sperm inactive (Fig. 1C, Extender part 1, deionized water part 0), but upon dilution (Fig. 1C, extender part 1, deionized water parts > 0) could activate sperm. Some BSMIS and FBS dilutions had activation rates comparable to tank water, while BSMIS 0.5 × (diluted with 1 part of deionized water) had significantly higher activation compared to tank water (p < 0.0012). HBSS and Iwamatsu solutions were not significantly better than tank water as activators at any dilution.

**Figure 2:**
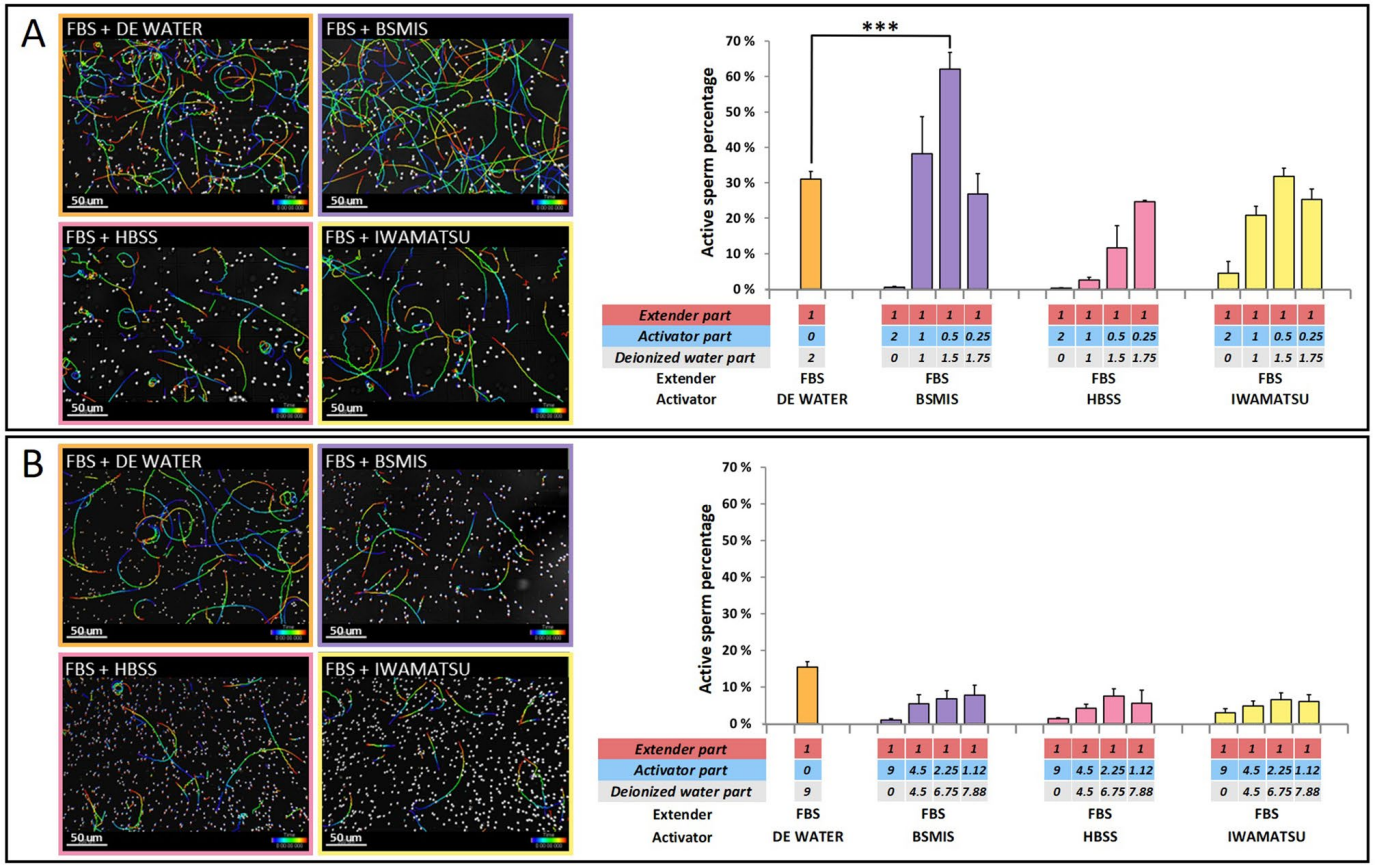
Fresh sperm activation using FBS as extender and different buffers for dilution. (**A**) Image tracking of sperm movements and quantification of directional sperm, using FBS extender solution mixed in a ratio of 1 to 2 with different activating solutions (1 volume of extender solution plus 2 volumes of activating solution at different dilutions). Ratios of extender, activator, and water for each mix are shown below each column. (**B**) Image tracking of sperm movements and quantification of directional sperm, using FBS extender solution mixed in a ratio of 1 to 9 with the different activating solutions (1 volume of extender solution plus 9 volumes of activating solution at different dilutions). Ratios of extender, activator, and water for each mix are shown below each column. Asterisks indicate solutions significantly more effective than tank water in activating sperm, based on a two-way ANOVA post hoc Dunnett’s test. *p < 0.05, **p < 0.01, ***p < 0.001. N = 3 biological replicates for each condition. (Image tracking of sperm movements were exported from Imaris, version 8.1, https://imaris.oxinst.com/).

FBS is also often used as an extender, which when diluted with other solutions activates sperm^17,19,20^. We therefore tested FBS buffer as extender, and added Iwamatsu, as well as BSMIS and HBSS at different dilutions for activation (Fig. 2A).

Among the combinations, we observed the highest activation using 1 part of FBS mixed with 2 parts of a solution of BSMIS 0.25 × (i.e. 1 part of FBS mixed with 0.5 parts of BSMIS and 1.5 parts of deionized water), reaching yields of > 60%. In contrast to medaka, FBS mixed with Iwamatsu did not give appreciable activation of *Nothobranchius* sperm. In another set of experiments, we increased the dilution ratio between the extender (FBS) and the activators to 1 plus 9 parts (Fig. 2B). However, the activation rates at these dilutions were much lower than in the previous tests (Fig. 2A), and most of the spermatozoa were vibrating or not moving at all.

### Cryoprotection, freezing and thawing

**Figure 3:**
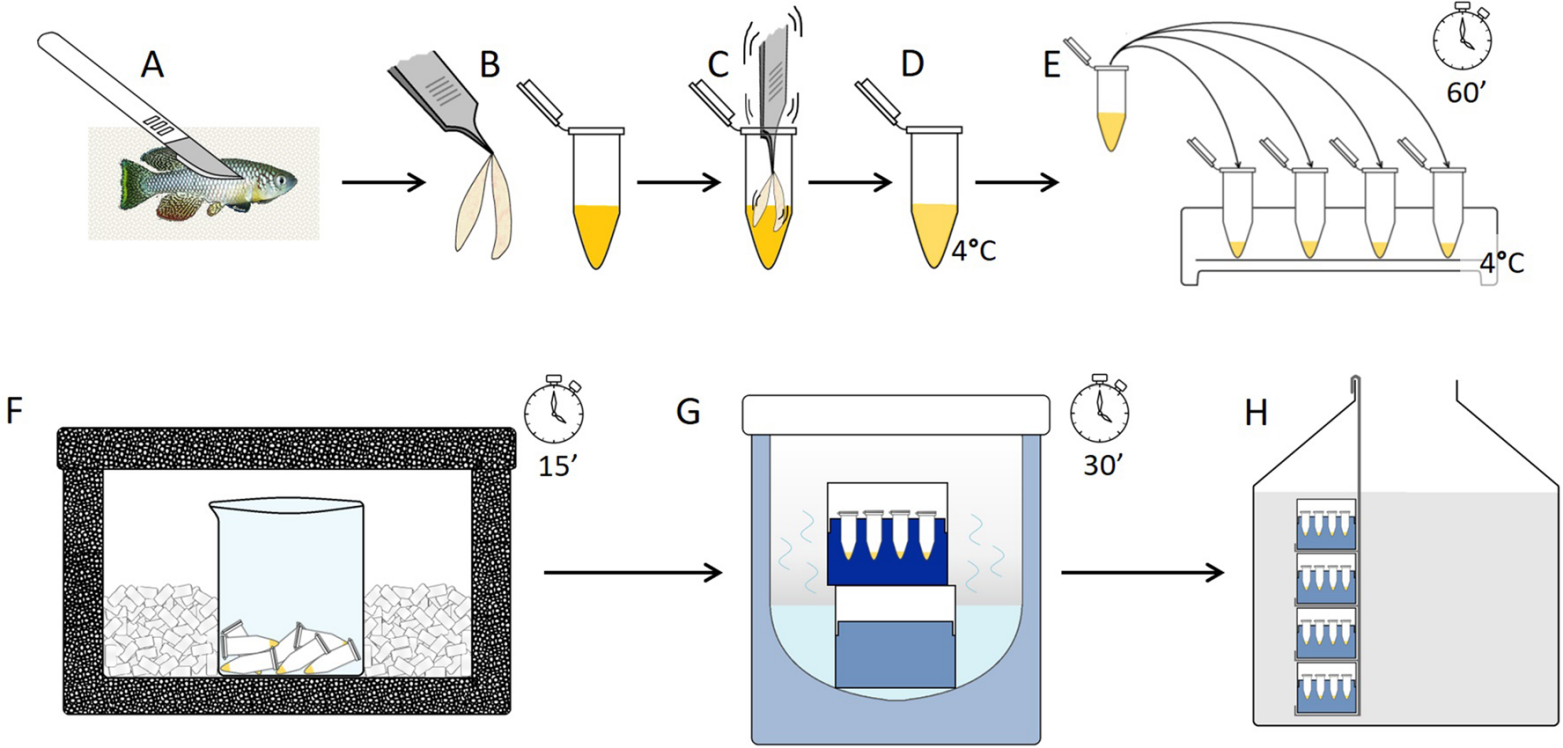
Schematic of sperm collection and freezing procedure. (**A**) Male dissection. (**B**) Gonad extraction. (**C**) Gonads are spun in extender plus cryoprotectant solution. (**D**) Cryoprotectant incubation with sperm cells at 4 °C. (**E**) Solution transfer in smaller aliquots to vials. (**F**) First freezing step setup with vials laying on the bottom of a glass beaker surrounded by dry ice in a closed styrofoam box. (**G**) Second freezing step setup with vials exposed to nitrogen vapor. (**H**) Long term storage of vials in liquid nitrogen. For a detailed explanation, see text.

For sperm to survive freezing and be kept long-term in a cryostatic condition, the extender has to be supplemented with a cryoprotectant. These chemicals surround sperm cells and prevent the formation of ice crystals that compromise membrane integrity, thereby protecting them from cryodamage^21^. The most commonly used cryoprotectants are DMSO, DMF, MetOH, glycerol, and DMA, with concentrations usually set around 10%^14,22^. Since FBS and BSMIS worked best as extenders (Figs. 1C, 2A), we performed our cryostatic experiments using these solutions, following the protocol outlined in Fig. 3 for cryopreservation.

**Figure 4:**
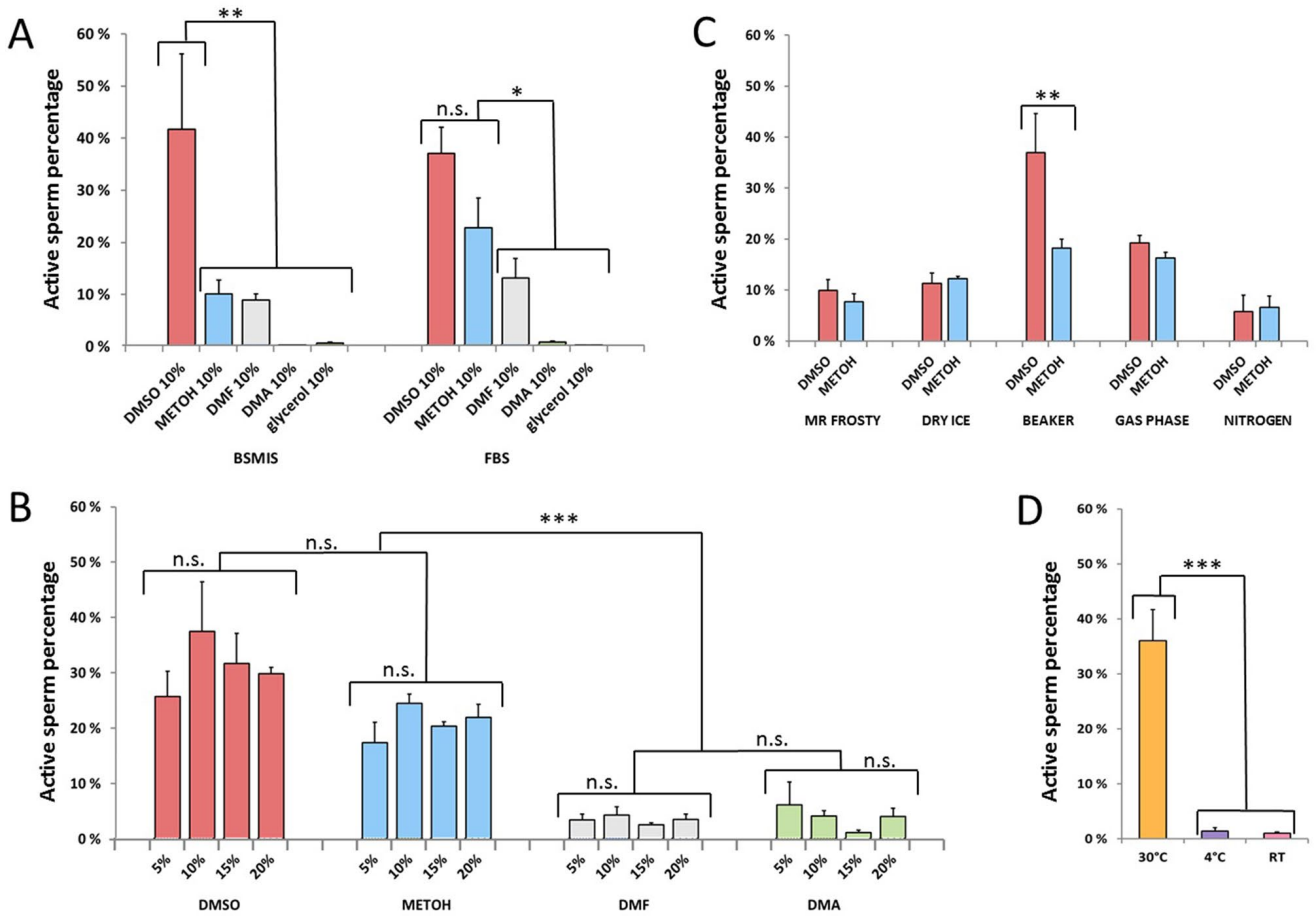
Cryoprotectants, cryopreservation and thawing efficiencies. (**A**) Effect of different cryoprotectants on sperm activation using BSMIS and FBS extender solutions. The Y axis represents the percentage of sperm able to reactivate upon thawing. Statistical analysis used is two-way ANOVA with multiple comparison Tukey’s post hoc correction. (**B**) Effect of different concentrations of cryoprotectants in FBS extender. Asterisks indicate significance levels of two-way ANOVA multiple comparison test with post hoc Dunnett’s correction. *p < 0.05, **p < 0.01, ***p < 0.001. (**C**) Various freezing set ups with different cooling rates applied to FBS extender plus 10% cryoprotectant. Asterisks indicate significance levels of two-way ANOVA multiple comparison test with post hoc Sidak’s correction. *p < 0.05, **p < 0.01, ***p < 0.001. (**D**) Various thawing methods (30 °C water bath, 4 °C refrigerator, RT, room temperature) with different thawing rates applied to FBS extender plus 10% DMSO. Asterisks indicate significance levels of one-way ANOVA multiple comparison test with post hoc Tukey’s correction. *p < 0.05, **p < 0.01, ***p < 0.001. N = 3 biological replicates for each condition.

Overall we found that sperm cryopreserved using 10% DMSO as cryoprotectant maintained the highest level of activation (Fig. 4A), followed by methanol (in FBS extender only). In BSMIS extender, DMSO performed significantly better compared to the other cryoprotectants (p < 0.0041). In FBS extender, DMSO and MetOH performed significantly better than the other cryoprotectants (p < 0.0359). DMF, Glycerol and DMA were ineffective at cryoprotection, resulting in immobile or inviable sperm upon thawing.

Several concentrations of cryoprotectants were further tested in combination with FBS. Sperm cryopreserved in FBS with 10–20% DMSO maintained comparably high activation ranging from 30 to 37% (Fig. 4B), dropping slightly at higher concentrations. Methanol was able to moderately protect sperm from cryodamage at several concentrations, giving sperm activation of ca. 20%. DMA or DMF gave less than 10% sperm revival at any concentration (Fig. 4B).

Since > 20% sperm survival and activation were observed in the samples with DMSO and methanol at 10%, we used these concentrations of cryoprotectants for further experiments.

The freezing procedure is also a crucial step for cryopreservation. Slow freezing rates can produce large ice crystals that damage cellular ultrastructure, whereas rapid freezing rates induce smaller intracellular ice crystals that are less likely to trigger damage^23^. We therefore used various setups (Mr. Frosty Freezing Container; glass beaker surrounded by dry ice; direct contact with dry ice; Dewar vessel partially filled with liquid nitrogen, where the Eppendorf tubes were placed in a box without direct contact with liquid nitrogen but exposed to nitrogen gas phase; direct liquid nitrogen contact) to establish different rates of freezing (Fig. 4C). Sperm vials frozen in a beaker surrounded by dry ice achieved the highest activation upon revival. Sperm cryoprotected with 10% DMSO had higher activation than those cryoprotected with 10% methanol (Fig. 4C), similar to above. Therefore, we selected DMSO as the final cryoprotectant and a beaker surrounded by dry ice for our cryopreservation protocol.

To assess the effect of different thawing rates on the frozen sperm, we warmed the vials at (1) 30 °C in a water bath, (2) room temperature or (3) 4 °C in the refrigerator, to achieve rapid, medium, or slow thawing rates, respectively. We then revived sperm using BSMIS 0.25 × and observed the percentage of activated sperm. We found that rapid thawing in a 30 °C water bath achieved the highest survival and activation of sperm (Fig. 4D).

### Egg fertilization and survival

**Figure 5:**
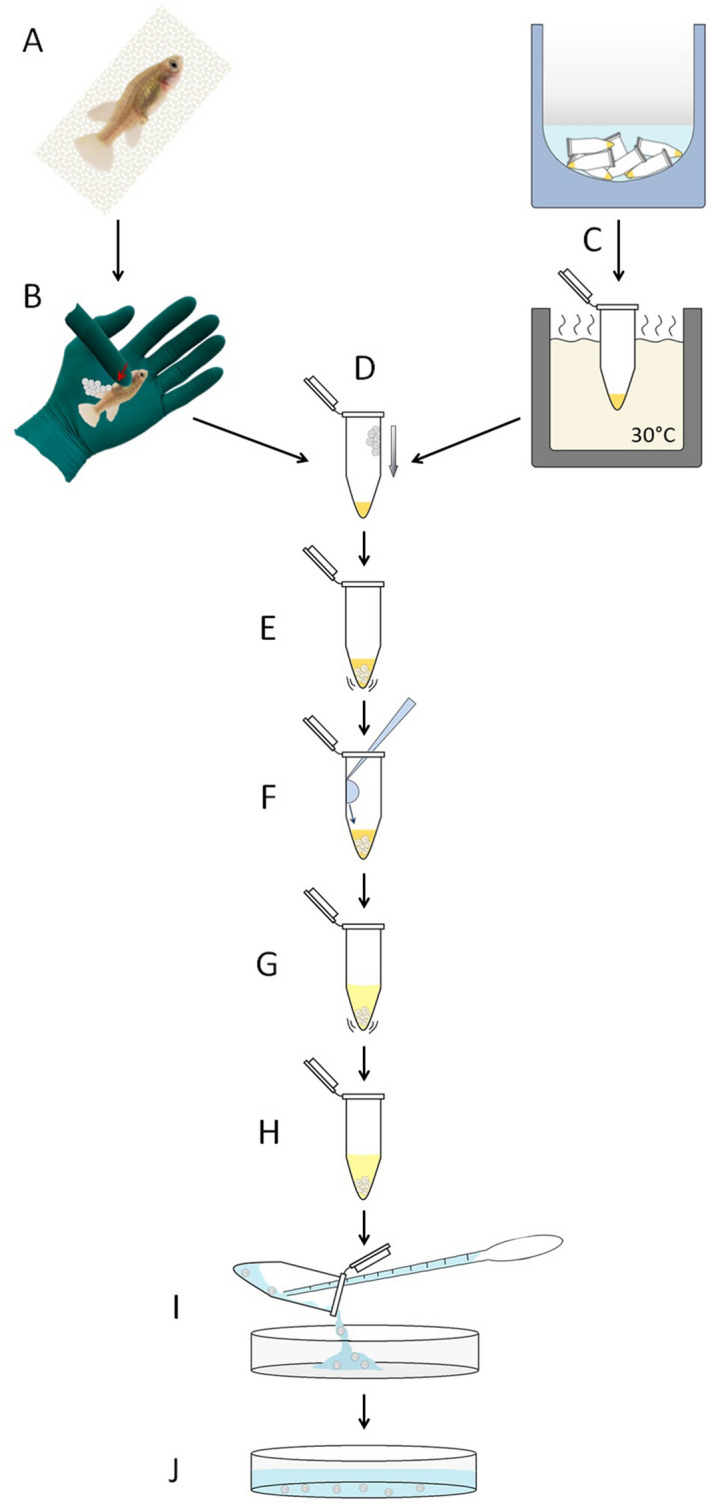
Schematic of sperm thawing and egg fertilization procedure. (**A**) Female preparation. (**B**) Egg collection through gentle abdominal squeezing. (**C**) Frozen sperm thawing in a water bath. (**D**,**E**) Eggs mixed with sperm-extender solution. (**F**) Activating solution addition and (**G**) mix. (**H**) Fertilization. (**I**) Egg recovery in a petri dish. (**J**) Fertilized eggs developing in a petri dish. For detailed explanation, refer to the protocol in S1 file and “Materials and methods”.

We next tested if the active sperm obtained with our protocol was able to fertilize eggs obtained from *N. furzeri* females, following the procedure outlined in Fig. 5.

**Figure 6:**
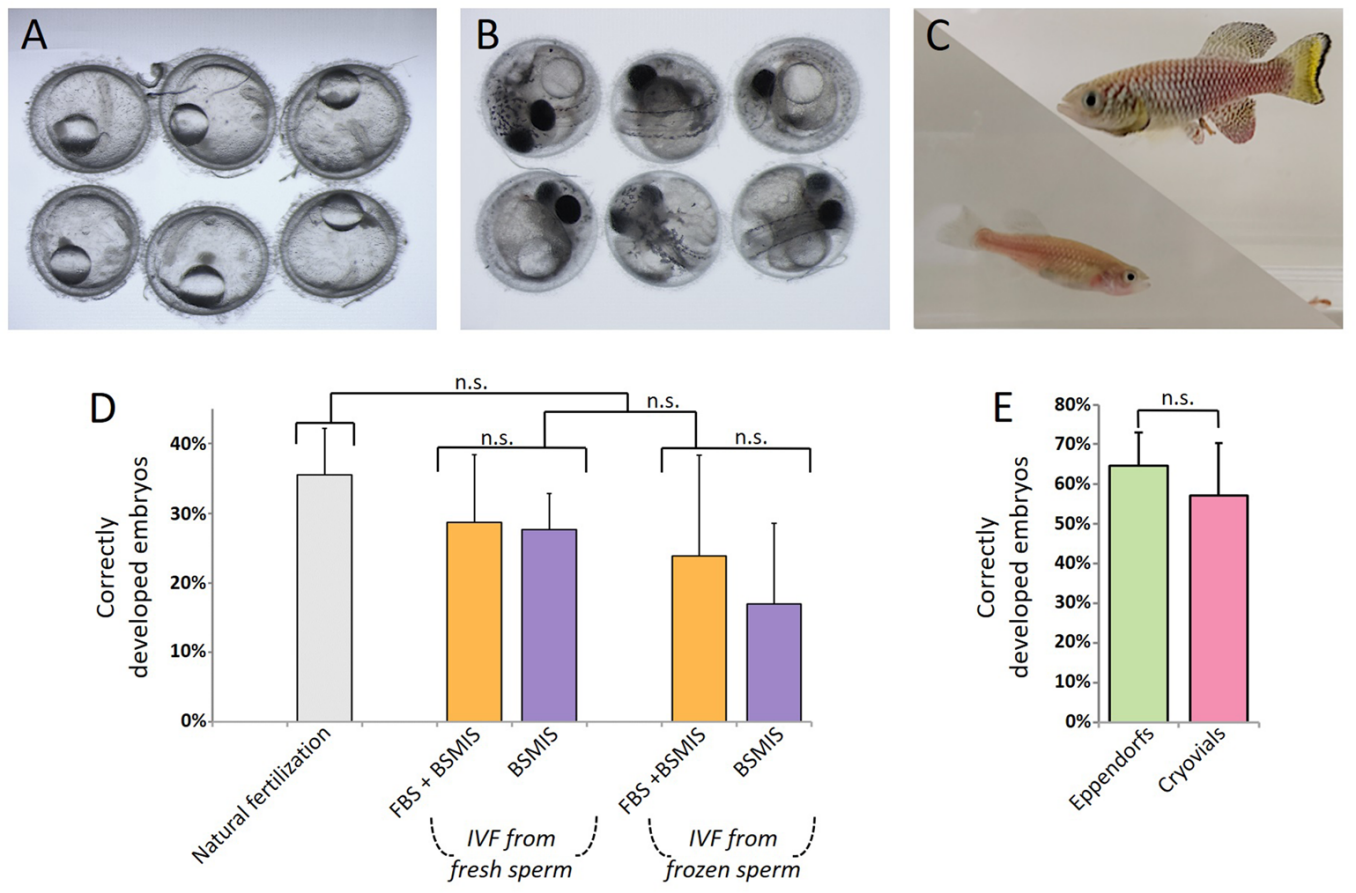
Fertilization rates and embryonic development. (**A**) Embryos fertilized with IVF using frozen sperm develop through diapause II/mid-somitogenesis stage and later (**B**), up to advanced developmental stage. (**C**) Adult fish derived from IVF embryos. (**D**) Fertilization efficiencies of natural breeding, IVF with fresh extracted sperm, and IVF with sperm frozen for 2 weeks. ANOVA post hoc Tukey’s test. N = 6, 8, 4, 10, 3 independent fertilization trials for each fertilization condition, respectively, in order of appearance in the graph. N = 1023, 440, 349, 485, 201 total eggs used for fertilization in the trials for each fertilization condition, respectively, in order of appearance in the graph. (**E**) Fertilization efficiencies of IVF using sperm frozen for 50 days in Cryovials or Eppendorf tubes. The egg pool used for fertilization derived from 10 weeks old females. Three independent trials were performed using different females and sperm aliquots. N = 45 total eggs used for cryovials (fertilized with 3 different sperm aliquots) and N = 52 total eggs for Eppendorfs (fertilized with 3 different sperm aliquots) tests. *t* test between the two groups results was not significant.

Eggs fertilized both naturally and in vitro were monitored until the stage of mid-somitogenesis (Fig. 6A) or later (Fig. 6B) and survival rates were scored (Fig. 6D). We found that under our conditions, rates of fertilization with frozen sperm were approximately 25% on average. This rate appears only slightly lower than that of fresh IVF or natural fertilization but is not significantly different due to high variability (Fig. 6D). Additionally this experiment was repeated again after 4 months using aliquots from our collection. Yields of fertilization were comparable to those achieved after 2 weeks freezing (Figure S1).

Several embryos were followed through development to post-somitogenesis (Fig. 6B), post-hatching and up to adulthood (Fig. 6C). The growth rates were normal, reaching maturity in 5–6 weeks, and no developmental defects were detected (n = 20). These fish were fertile and able to produce viable embryos (n = 70).

In a final experiment, we used the sperm previously extracted and frozen in Cryovials and Eppendorf tubes for 50 days to fertilize eggs of 10 weeks old females. In this experiment we used FBS as extender and BSMIS 0.25 × in deionized water as activator. Results were comparable between the two groups (Fig. 6E), suggesting that both tube types are suitable for short-medium term sperm storage. The long term storage (years) remains unexplored.

A detailed protocol for the entire procedure of egg in vitro fertilization is found in the Supplemental Information.

### Cortical reaction

**Figure 7:**
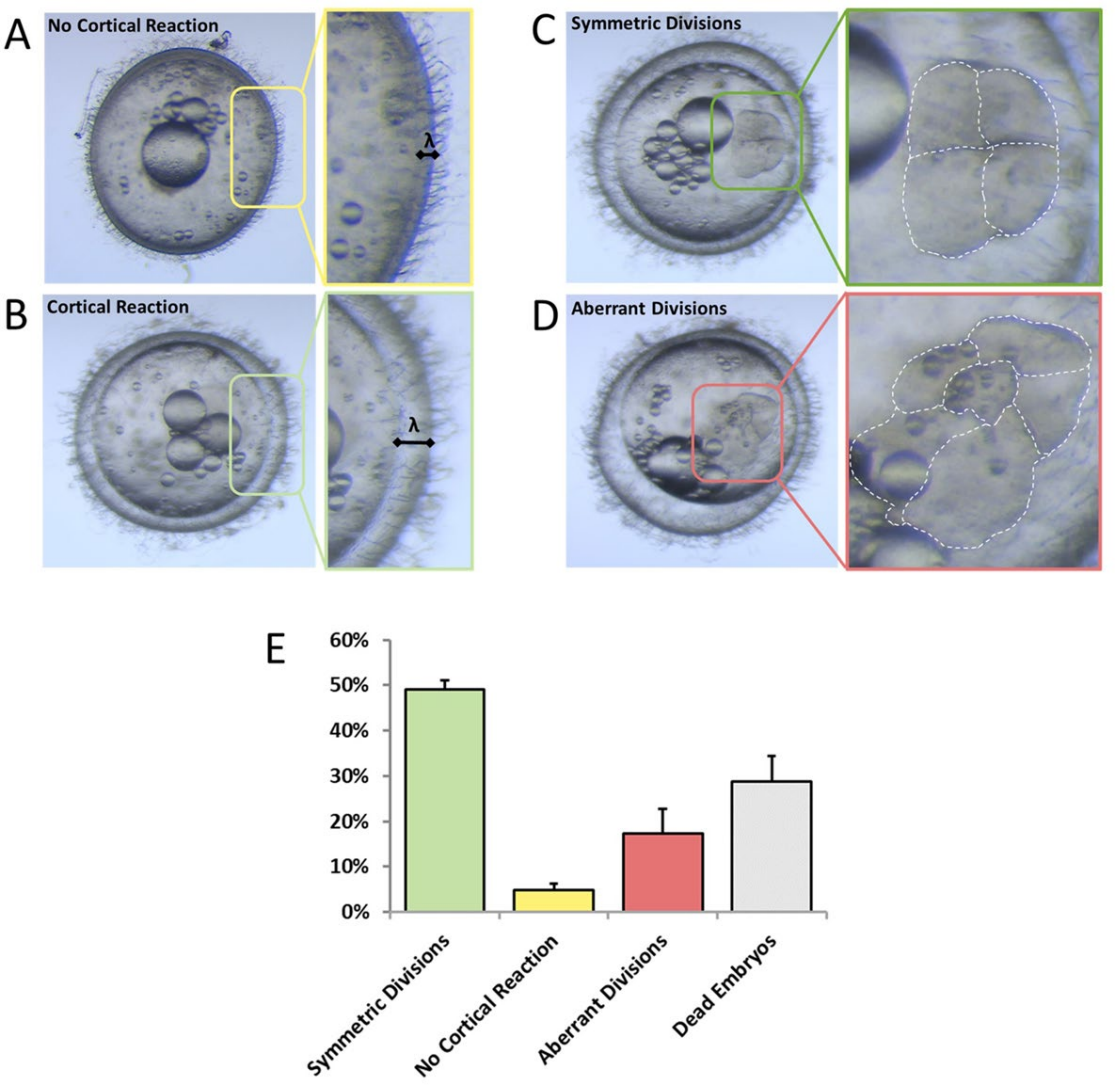
Cortical reaction and cell division in fertilized and unfertilized eggs. (**A**) Oocytes that do not undergo the cortical reaction display closely apposed chorion membrane and yolk (λ represents the average distance of 35.0 ± 10.6 µm, N = 8). (**B**) Embryo in which the cortical reaction has occurred properly (λ represents the average distance, of 87.6 ± 11.2 µm, N = 14). (**C**) Embryo undergoing correct cell division at the 4 cell stage. (**D**) Pseudoembryo undergoing aberrant divisions. (**E**) Average embryonic phenotypic distribution. N = 4 independent natural fertilizations with 470, 332, 101, 612 eggs collected, respectively. The graph represents the average of the 4 different collections.

Interestingly, we noticed that upon contact with the aqueous medium, eggs could spontaneously undergo the cortical reaction, even in the absence of sperm, as indicated by an increase in the distance (λ) between the yolk and chorion membrane from 35.0 ± 10.6 µm to 87.6 ± 11.2 µm (Δ = 52.7 ± 10.9 µm) (Fig. 7A,B).

Under conditions of natural fertilization, a fraction (10–25%) of eggs underwent spontaneous cortical reaction, while a smaller (1–10%) fraction of eggs remained blocked in the pre-cortical reaction stage (Fig. 7E, yellow bar). Eggs that underwent a spontaneous cortical reaction without fertilization, which we term “pseudoembryos,” displayed abnormal cell cleavages (compare Fig. 7C and D), with cells of odd number, irregular size and shape during the early cleavage stages. In addition these embryos showed errant development and embryonic death in the first 10 days (Fig. 7E, salmon bar). Eggs that did not carry out the cortical reaction were not fertilized, but evidently not all eggs that carried out the cortical reaction were fertilized either. Thus the cortical reaction itself does not necessarily indicate fertilization success.

## Discussion

In this study, we worked out procedures for sperm activation, cryopreservation and in vitro fertilization in the species *N. furzeri,* aiming to establish protocols to facilitate line maintenance and space usage of this fish. Concentrating on commonly used buffers and testing them in several dilutions and combinations, we found that *N. furzeri* requires a specific protocol tailored for this species. We narrowed down the viable extender solutions to FBS and BSMIS and activators to BSMIS 0.25 × and 0.5 ×. Cryoprotectants 10% DMSO and MetOH best prevented cryodamage and preserved sperm viability. Moderate freezing rates in a beaker using dry ice, and rapid thawing at 30 °C produced sperm that could be readily activated. Our IVF method resulted in fertilization rates that were comparable between frozen sperm, fresh sperm and natural fertilization, hovering around 20–30%. Cryopreserved sperm was equally active at least 4 months later.

Nevertheless, there are several parameters that we did not explore and possible limitations of our study. First and foremost, we do not know the maximal length of time that sperm can remain well preserved under these conditions. Based on other reports^24^, we anticipate preservation could last many years. We used sperm densities of ca. 60,000/μl and male age of 9–11 weeks (see “Materials and methods”); we did not systematically optimize these parameters. Another limitation of our study is that males were sacrificed in order to retrieve sperm from the testes. Future techniques could be developed to collect sufficient sperm from live animals. Finally, sperm preservation only represents solving half the problem. In the future, it would be extremely useful to develop techniques for egg preservation as well.

Currently most *N. furzeri* lines are kept in embryonic diapause for long-term maintenance. This still remains a very useful approach because it preserves lines in the form of the embryo, not as gametes, and does not require freezers or liquid nitrogen systems that can break down. Though embryos reportedly can be maintained in diapause as much as 3 years^25^, it is still likely less time than cryopreservation of sperm. Maintaining embryos in diapause is also more time consuming and takes up greater space. Whether animals in embryonic diapause can also be cryopreserved is also unknown. In any case, sperm cryopreservation techniques and IVF can complement existing techniques of diapause preservation.

One of the unexpected observations we made in the course of our studies is that a considerable number of oocytes undergo the cortical reaction even in the absence of sperm, under conditions of IVF or natural fertilization. Such “pseudoembryos” displayed abnormal development. Though the mechanism underlying spontaneous cortical reaction remains unknown in this species, in other species it is thought to be related to the degree of egg maturation^26,27^. Whether conditions can be improved to minimize such events to increase the fraction of fertilizable oocytes is another parameter that deserves future attention.

## Conclusion

In sum, we worked out conditions feasible for sperm cryopreservation and in vitro fertilization in *Nothobranchius furzeri*. Our protocol provides useful techniques that can enhance existing approaches for line maintenance and husbandry, which should greatly facilitate the use of *Nothobranchius furzeri* as a model organism.

## Materials and methods

### Ethical approval

All fish husbandry was performed in the fish facility of the Max Planck Institute for Biology of Ageing (Permit Nr. 576.1.36.6.G12/18 Be). Animal experiments were approved by the Max Planck Institute for Biology of Ageing Institutional Animal Welfare Body (RB.16.005) and complied with national legislation and the European directive 2010/63/EU. The study was overseen and reported to the appropriate authority, the Veterinarian State Office Cologne. The study is reported in accordance with ARRIVE guidelines.

### Fish husbandry and sample collection

All adult fish used were *Nothobranchius furzeri* belonging to the GRZ strain and were raised singularly in 2.8 L tanks from the second week of life. Water parameters were pH: 7–8; Kh: 3–5; T: 27 °C. A volume equal to 10% of the water in the system was automatically replaced with clean water every day. Fish were raised in 12 h of light and 12 h of darkness. Fish were fed a mixture of Chironomus larvae and “Premium Artemia Coppens” twice a day. Natural breeding events took place in 8 L tanks with 1 male and 3–5 females. To initiate breeding, one box (9 cm × 9 cm × 4 cm) half full of river sand (diameter < 0.2 mm) was put on the bottom of the tank. The boxes were put in the fish tanks for 2–3 h during which the average number of eggs laid by each female in the sand was between 5 and 30. All the fish used for experiments were from 9 to 11 weeks old. Male body length and weight at 11 weeks was approximately 4.3 ± 0.2 cm and 1.23 ± 0.12 g, respectively (N = 3). Female body length and weight at 11 weeks was approximately 3.27 ± 0.175 cm and 0.42 ± 0.037 g, respectively (N = 6). The length and weight of paired gonads arms extracted from 11 week old males were approximately 0.73 ± 0.058 cm and 16.77 ± 1.47 mg, respectively (N = 3).

### Gonad extraction and sperm collection

Males were euthanized with tricaine methanesulfonate (Sigma #E10521, 0.5 mg/ml) for 10 + minutes, until movement and breathing ceased. Fish were dried using tissue paper to prevent spontaneous sperm activation due to the presence of water and decapitated. The abdomen was cut open with scissors, the organs removed and the testes flanking the swimming bladder were gently extracted using a forceps. Gonads were placed in an Eppendorf tube containing 500 μl of extender solution, held with forceps and spun back and forth for 1 min. After the sperm was released into the solution, the gonads were removed from the Eppendorf tube. On average around 30 million sperm (between 7.5 million and 100 million) were released. This value was estimated by counting the sperm cells of several sperm-extender aliquots in a 3 × 3 square field of a Burker chamber. The initial total sperm value was calculated multiplying the number obtained by the dilution factor.

### Fresh solutions trials (Fig. 1)

One gonadal arm of the testes was placed in 250 μl of solution (tank water, DE water, BSMIS, HBSS, FBS, Iwamatsu solution) at various dilutions (1 ×, 0.5 ×, 0.25 ×, 0.125 ×) and the sperm was released inside by shaking for 5–10 s. Immediately afterwards, gonads were removed from the medium, 10 µl of the mixture was put in a hemocytometer chamber and sperm movements were video-recorded under a microscope. The videos were subsequently analyzed using Imaris and the percentage of directionally moving spermatozoa measured (Fig. 1A–C). Each trial combination was repeated at least 3 times with gonads derived from different fish.

BSMIS (1 ×): (75 mM NaCl, 70 mM KCl, 2 mM CaCl_2_, 1 mM MgSO_4_, 20 mM Tris pH 8.0).

HBSS: (Gibco #14025-092).

FBS: (Gibco #10270-106).

### Extender solution sperm activation trials (Fig. 2)

One gonadal arm of the testes was extracted and shaken in extender solutions as described above. One volume (3 μl) of sperm-extender mixture was mixed with two (6 μl) or nine volumes (27 μl) of activator solutions and immediately transferred to the hemocytometer chamber under the microscope for video recording. Each trial combination was repeated at least 3 times with gonads derived from different fish shaken in the extender.

### Imaging and video acquisition

For sperm visualization, we used a Zeiss Imager Z1 microscope and recorded videos with a Zeiss Axiocam 506 mono, using parameters of binning 3 × 3, resolution 2752 × 2208 pixels, and the ZEN 2.3 Pro software. Each video was recorded for 8 s.

### Imaris conversion and sperm motility analysis

Videos were imported in Imaris 8.1 and frame time points rescaled by 10 times. Black and white colors were inverted in order to visualize white dots over a black background. The particle tracking function was set to identify all sperm with a diameter of ± 2.9 μm. Movements were tracked with the autoregressive motion algorithm function, set with maximum distance of 25 μm and a maximum gap size of 5. Statistics related to track displacement length were exported as Excel file and analyzed. As a cutoff, we set a displacement length of > 15 μm to distinguish directionally traveling sperm from non-active or vibrating sperm. In the case of general drift of the whole sample size we increased the cutoff to > 35 μm or > 50 μm (in the case of very strong drift).

Raw data depicting track displacement length realized with Imaris were exported as an Excel chart. Data related to all the experiments were pooled in a unique Excel file, containing 3 biological replicates for each individual trial or combination. Total data were used to calculate averages, standard deviations and standard errors. Graphs were produced from these data using Excel.

### Sperm collection and freezing procedure (Fig. 3)

The selected extender (BSMIS or FBS) was mixed with 10% solution of DMSO, DMF, MetOH, DMA or glycerol, in different tubes. Sperm was released in these pre-mixed extender-cryoprotectant solutions (Fig. 3C) and the mixture was incubated for 1 h at 4 °C (Fig. 3D), allowing the cryoprotectants to be evenly absorbed by the sperm cells. Samples were then aliquoted in 60 μl volumes (Fig. 3E), frozen in dry ice (Fig. 3F), and nitrogen gas phase in sequence (Fig. 3G), and stored in liquid nitrogen (Fig. 3H). After 24–48 h of freezing, the sperm were revived through rapid thawing in a 30 °C water bath (see optimization below) and activated by the addition of 2 volumes of BSMIS (0.25 × in the case of FBS extender and 0.5 × in the case of BSMIS extender).

### Cryoprotectant trials (Fig. 4A,B)

For each biological replicate the whole testes were extracted from a male and were shaken in 500 μl of different extender solutions (FBS or BSMIS). In total we used 3 biological replicates for each extender combination. Mixtures were aliquoted in smaller volumes (60 μl) and different cryoprotectants (DMSO (Sigma), DMF (Carl Roth), Methanol (Optima), DMA (Sigma) or glycerol ≥ 99.5% (Sigma) were added in different concentrations in each Eppendorf and immediately mixed. A volume equal to 3 μl from each sample was taken and mixed with an activation solution to check the sperm mobility, following the procedure previously described. The rest of each mix was frozen following the cryopreservation procedure. Sperm survival was checked after 2 weeks upon thawing (see below).

### Freezing trials (Fig. 4C)

Both gonads from the same individual were placed in 500 μl FBS solution and shaken for sperm release. A volume equal to 60 μl of the resulting mix was mixed with 10% DMSO or 10% methanol in Eppendorf tubes (or Cryovials, Sarstedt #72.692.005) and incubated for 1 h at 4 °C. Vials were distributed to (1) Mr. Frosty Freezing Container (estimated − 1 °C per min), (2) a glass beaker surrounded by dry ice (estimated − 20 °C per min), (3) direct contact with dry ice (estimated − 50 °C per min), (4) a Dewar vessel partially filled with liquid nitrogen, where the Eppendorf tubes were placed in a box exposed to nitrogen gas phase (estimated − 100 °C per min); and 5) direct liquid nitrogen contact (estimated − 200 °C per min) (Fig. 4C). Cooling rates were estimated using a digital thermometer with a detection range − 50 °C to + 110 °C with the probe inserted in the tube. Cooling rate was calculated by measuring the temperature difference between room temperature and − 50 °C, divided by the time required to reach − 50 °C. Fifteen minutes after the temperature of − 50 °C or below was reached, vials were removed from their freezing setups and placed in liquid nitrogen overnight. After one day, frozen sperm samples were revived using BSMIS 0.25 × and monitored for activation.

### Thawing and sperm revival (Fig. 4D)

Frozen samples were thawed directly from liquid nitrogen into a 30 °C warm water bath until the ice completely melted. The procedure usually took 1 min or less. One volume (3 μl) of thawed sperm mixture was mixed with two volumes (6 μl) of BSMIS 0.25 × and pipetted into a hemocytometer chamber to record sperm movements.

### Cryopreservation (Fig. 5)

Extender-sperm-cryoprotectant mixtures were incubated in 1.5 ml Eppendorf tubes for 1 h at 4 °C. The mixtures were distributed in Eppendorf tubes at a volume of 60 μl each, the tubes were closed and gently placed at the bottom of a glass beaker sitting in a styrofoam box filled up to 10 cm with dry ice. After 15 min, Eppendorf tubes were quickly moved into a rack in a Dewar cryovessel and exposed to nitrogen vapors for 30 min. Samples were moved directly into liquid nitrogen tanks for long term storage.

### In vitro fertilization by thawed sperm (Figs. 5, 6)

To assess natural and in vitro fertilization rates, the same males were used for direct comparisons. Each fertilization involved 5 females and 2 males per tank in which animals were allowed to breed naturally for 2 days. Breeders were typically 9–11 weeks of age. The eggs generated from the natural breeding were collected and their survival rate was monitored until mid-somitogenesis. After the natural breeding, the same males were separated from the females, isolated for 2 days, and then sacrificed for gonad extraction and sperm retrieval (Fig. 3A,B).

The obtained sperm was mixed with FBS or BSMIS buffer containing 10% DMSO. Half of the sperm-extender-cryoprotectant mixture was directly activated (with 0.25 × or 0.5 × BSMIS) and used for the fertilization of the eggs (Fig. 5D–J) while the other half was frozen and cryopreserved (Fig. 3). A subset of the sperm was also frozen in Cryovials with the same shape as the 1.5 ml Eppendorf tubes. After 2 weeks, frozen sperm were thawed, activated and used to fertilize another pool of eggs derived from 10 weeks old females, following the same procedure (Fig. 5). Sperm concentrations used for fertilization were on average 60,000/μl, a value estimated by counting the number of sperm in a 3 × 3 square field Burker chamber.

For IVF, females were anesthetized in a Tricaine methanesulfonate solution (0.5 mg/ml) and carefully dried of residual water to prevent spontaneous egg cortical reaction or activation (Fig. 5A). Placing the fish on an open hand, gentle pressure was applied with a finger on the abdomen, pushing gently from the middle toward the anus (Fig. 5B). Typically 5–35 eggs were expelled. Eggs were collected using forceps and placed into an Eppendorf tube containing the extender-sperm solution (Fig. 5D). In the case of frozen sperm, the tube was thawed immediately before fertilization in a 30 °C water bath (Fig. 5C). These steps were best performed by two people, with one person thawing the sperm while the other person expelled eggs from the female. Eggs were placed at the edge of the tube and gently pushed to the bottom (Fig. 5D)*.* No more than 35 eggs were placed in 60 μl aliquots of sperm-extender. Once the eggs were immersed, the tube was gently flicked for 10–20 s (Fig. 5E), allowing homogeneous distribution of sperm around the eggs. The activator solution was then pipetted into the tube, letting drops slide over the tube’s wall (Fig. 5F) and then mixed with the extender by gently flicking the tube for 20–30 s (Fig. 5G). Activated sperm was incubated with the eggs for 10 min, with the tubes standing open on a bench at room temperature (Fig. 5H). At this step, 10 μl of the mixture was examined under the microscope to evaluate sperm motility. To avoid damage due to DMSO toxicity, the eggs were transferred after 10 min by pipette to a petri dish and methylene blue buffered tank water (Fig. 5I). The water was replaced twice and the petri dish incubated at 28 °C (Fig. 5J).

To approximate the fertility rate, we counted the total number of embryos that developed until mid-somitogenesis (Fig. 6A) over the total number of eggs used for fertilization (Fig. 6D).

### Embryo phenotype assessment and cortical reaction (Fig. 7)

Ten breeding tanks were set up with 1 male and 4 females each. Fish were allowed to breed for 3–4 h and then eggs were collected and cleaned of empty shells or dead eggs and allowed to develop in an incubator at 28 °C for 6 h. Embryos were then carefully examined under a Leica M80 stereomicroscope to assess morphology, cortical reaction, cell shape, and number. Four independent breedings were performed with at least 2 days between them. Each breeding event yielded 470, 332, 101, 612 embryos, respectively. Data regarding the percentages of the various phenotypes were averaged between the 4 collections to produce the graph in Fig. 7.

### Statistics

Statistics were calculated using GraphPad Prism. For pairwise comparisons, the *t* test was used, while multiple comparisons were done by ANOVA. The specific type of ANOVA (one-way or two-way) and post hoc test used are indicated in figure legends.

### Images acquisition and enhancement

Tracking images were acquired from videos using Imaris snapshot function. Brightfield images were acquired using a Leica M80 microscope equipped with a Leica MC170 HD camera. Images were enhanced in brightness, contrast and saturation using GIMP to improve the visual quality. Quantifications were not affected by imaging quality enhancing modifications.

Graphics and drawings were realized using Paint, GIMP and Power point.

## Supplementary Information

Supplementary Information.

## Data Availability

All data generated or analyzed during this study are included in this published article (and its Supplementary Information files). The datasets generated during and/or analyzed during the current study are available from the corresponding author on reasonable request.

## Abbreviations

HBSS: Hank’s Balanced Salt Solution
BSMIS: Buffered sperm motility-inhibiting solution
FBS: Fetal bovine serum
DMSO: Dimethyl sulfoxide
DMF: Dimethylformamide
MetOH: Methanol
DMA: Dimethylacetamide
IVF: In vitro fertilization
