# Supplementary material for: Sperm cryopreservation and in vitro fertilization techniques for the African turquoise killifish Nothobranchius furzeri
Source: Sci Rep. 2021 Aug 25;11:17145. doi: 10.1038/s41598-021-96383-8 (PMC8387425; doi:10.1038/s41598-021-96383-8)
Supplement: Supplementary file 1 — Supplementary Information. [file 41598_2021_96383_MOESM1_ESM.docx]

**Supplemental Information, Dolfi et al.**

**
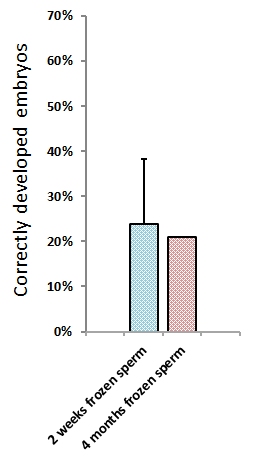
**

**Figure S1. Sperm resilience in FBS + 10% DMSO extender.** Fertilization efficiencies of IVF with sperm frozen for 2 weeks or 4 months. N = 10 independent fertilization trials (485 total eggs used)  with sperm frozen for 2 weeks in FBS + 10% DMSO and N = 1 fertilization trial (52 total eggs used)  with sperm frozen for 4 months in FBS + 10% DMSO.

**Protocol for cryopreservation and *in vitro* fertilization.**

I. Protocol for cryopreservation:

1. Aliquot 540μl FBS (Gibco #10270-106) and 60μl DMSO (Sigma #D4540) into a 1.5ml Eppendorf tube or similarly shaped cryovial (Sarstedt #72.692.005) to prepare the extender cryosolution. Mix well.
2. Select a male specimen, preferably 9-11 weeks of age. To euthanize, treat with tricane methanesulfonate (0.5mg/ml, Sigma #E10521) in a 0.5l box until breathing stops.
3. Dry the male carefully, decapitate, make an incision in the abdomen with a scissors, and extract the gonads.
4. Tightly grasp the gonads with a forceps and shake them vigorously inside the extender cryosolution prepared in step 1 for 1 minute. Use only one gonad if the fish is old (>13 weeks) and the gonads are large.
5. Remove the residual gonads from the solution. Check that the solution is opaque. If not repeat step 4.
6. Keep the sperm/cryosolution on ice for 45 minutes.
7. For quality check, take 15μl from of the sperm/cryosolution and gently mix with 30μl of BSMIS^1^ 0.25X on a glass slide or in a petri dish to make a 45μl drop. Avoid air bubbles! Check the edges of the drop (the contrast in the middle is usually not high enough for sperm detection) to assess sperm activation under a microscope. Look for directional movement.
8. Gently resuspend the sperm/cryosolution on ice and make 10 aliquots of 60μl each.
9. Let the aliquots sit 15 minutes on ice.
10. Place a glass beaker into a styrofoam box and fill the empty space with dry ice.
11. Gently place the aliquots at the bottom of the beaker and close the lid on the styrofoam box. Let the solution freeze for 15 minutes.
12. Prepare a 2l size Dewar cryovessel having liquid nitrogen filled approximately to a height of 4 cm and place an empty cryobox in it.
13. Place the cryotubes in another cryobox and place this box above the other in the vessel, letting only the nitrogen vapour reach the tubes.
14. After 30 minutes, immerse the cryotubes into the liquid nitrogen.
15. Cryotubes are now ready for long term storage in a liquid nitrogen tank.

II. Protocol for IVF:

(from here on, a second person could be required to facilitate the speed and smoothness of each step)

1. Prepare a 30°C water bath.
2. Anesthetize a female using tricaine (0.5mg/ml) for 2 minutes.
3. Carefully dry the female with paper towel.
4. Wearing latex gloves, lay the female on an open palm and gently apply pressure on the abdomen with a finger, moving it down toward the anal fin, until eggs come out.
5. As the first egg emerges, place one of the sperm/cryoextender tubes immediately in the water bath for 30 sec to 1 min until completely thawed.
6. Gently grab all the eggs at once using a forceps and position them to the bottom of the thawed tube. Put no more than 35 eggs in the tube.
7. Gently shake the tube for 10-20 seconds and let the solution homogeneously distribute throughout the eggs.
8. Add 120μl of BSMIS^1^ 0.25X to the edge of the tube and let the solution slide to the bottom.
9. Gently mix the solution for 20-30 seconds.
10. As a quality check, take 15 μl from the solution and put on a glass slide or petri dish. Check the edges of the drop for sperm activation under a microscope.
11. Allow fertilization to occur for 10 minutes at room temperature.
12. Wash the fertilized embryos out from the tube to a petri dish using aquarium water.
13. Change the water twice and incubate embryos at 28°C.

^1^BSMIS 1X = 75mM NaCl, 70mM KCl, 2mM CaCl_2_, 1mM MgSO_4_, 20mM Tris pH8.0
